# Acculturation Experiences of Adolescents and Nonsuicidal Self-injury Behaviour: An Interpretative Phenomenological Analysis

**DOI:** 10.1007/s40653-025-00712-2

**Published:** 2025-05-16

**Authors:** Xénia Volovik, Lan Anh Nguyen Luu, Eszter Petra Frank-Bozóki, Judit Balázs

**Affiliations:** 1 Institute of Psychology, Eötvös Loránd University, Budapest, Hungary; 2 Doctoral School of Psychology, Eötvös Loránd University, Budapest, Hungary; 3 Institute of Intercultural Psychology and Education, Eötvös Loránd University, Budapest, Hungary; 4 Oslo New University College, Oslo, Norway

**Keywords:** Acculturation, Mental health, Impact of immigration, Self-harm, Quality of life, Immigration experience

## Abstract

Several studies have shown that the process of immigration and the accompanying stress of acculturation and adaptation are risk factors for the appearance of mental disorders in adolescents. To explore the adaptation and acculturation experience of youths reporting nonsuicidal self-injury behavior and mental health difficulties. Additionally, the study seeks to better understand these adolescents’ stressful experiences and strategies for coping. To answer the research questions, Russian-speaking adolescent immigrants were included in the study in Hungary. The immigrant adolescents were all from the former Soviet Union. We did not consider cultural background factors other than the use of the mother tongue. Adolescents who had experienced at least one episode of nonsuicidal self-harm or mental difficulties were included. The Mini International Neuropsychiatric Interview for Children and Adolescents, the Deliberate Self-Harm Inventory, and interviews related to the experience of immigration were analyzed using interpretative phenomenological analysis. The interviews were completed in 2018–2019, before the the COVID pandemic and the Russian—Ukrainian war. Altogether 5 adolescents were included. The experiences were organized around six topics. Through the themes presented, the stressors of immigration and the adolescents' attempts to cope with them are shown. The experiences that cause stress are wide-ranging and long-lasting, with the potential to impact mental health. In terms of acculturation, adolescents aspired to assimilation but were forced into separation due to the absence of a common language. From a mental health point of view, the potentially stressful circumstances are embedded in a context, which, if well understood, can be targeted with culturally sensitive stress prevention programs. Recommendations are made based on the results.

## Introduction

### Immigration and Mental Health

In today’s globalized world, immigration affects many families and communities (World Health Organization, 2021). With increasing numbers of immigrants worldwide, it is important to investigate the accompanying processes. According to the World Health Organization (WHO), which focuses on the general guidelines for the mental health of immigrants- immigrants belong to an at-risk group in terms of mental health, since acculturation and discrimination are potentially stressful and challenging experiences (World Health Organization, 2021).

Clinical studies and quality of life research conducted among adolescent immigrants show an inconsistent picture (Basu et al., 2022). While some of these studies have confirmed the more frequent occurrence of mental disorders and suicide risk and the experience of a lower quality of life among immigrants, other research has found no significant differences between the immigrant and host population (Belhajd et al., 2014; Amiri, 2022; Errazuriz et al., 2023). This inconsistency may stem from the different characteristics of the studied groups and host countries (Duinhof et al., 2020; Rotsika et al., 2016)—for example, the cultural background and family circumstances of the host population and the immigrants (Kim et al., 2018; Mirsky, 1997; Nesterko et al., 2018). In studies conducted with Russian-speaking adolescent immigrants, the negative impact of immigration on mental health was frequently documented (Mirsky, 1997; Nesterko et al., 2018; Ponizovsky & Ritsner, 1999, Knaifel et al., 2023, Stasulane, 2024). Higher risk of suicide was found in a study from Israel among Russian-speaking immigrants (Ponizovsky & Ritsner, 1999, Mirsky et al., 2011).

The presence of protective factors against migration-related psychosocial stressors may play an important role in mental health. Thus, for example, Miller et al.’s (2006) study of immigrants to the United States from the former Soviet Union indicated that higher levels of English language proficiency were associated with lower depression scores. Similar results were found for immigrants of other origins (Montemitro et al., 2021) High-quality social and family relationships, smaller cultural distance, and access to learning and job opportunities can likewise be considered as protective factors. Psychological adaptation—that is, low levels of anxiety and positive emotional states (coping and resilience)—contributes to successful integration,^1^ which is indicated by a better quality of life on the one hand, and sociocultural adaptation, which means having the necessary knowledge to manage comfortably in everyday life, on the other (Güngör & Perdu, 2017; Shekriladze & Javakhishvili, 2024; Ward, 2013).

### Russian Speakers in Hungary

Although there are no reliable data on the number of Russian speakers in Hungary, estimates range between 20,000 and 50,000 (Bendarjevskiy, 2011). According to Hungary’s Central Statistical Office (KSH) (2018), the number of Russian citizens in Hungary may currently be around 5,000, including around 300 adolescents between the ages of 15 and 19. These numbers most likely do not reflect reality: there are probably far more Russian speakers in Hungary, coming from the countries of the former Soviet Union (Bendarjevskiy, 2011).

Regarding the historical background, a number of Russian speakers came to Hungary at around the time of the First and Second World Wars (Bendarjevskiy, 2011; Polyan, 2004). Due to the Soviet occupation and the events of 1956, the reception of Russian speakers was associated with negative feelings and mistrust, which had an impact on the integration of those who settled (Bendarjevskiy, 2011). Language difficulties made integration even more difficult (Bendarjevskiy, 2011). Following the change of political regime in 1989, a larger wave of Russian-speaking immigrants arrived in Hungary for business purposes, but due to the mentioned language difficulties and mistrust, as well as other factors, they did not stay long term (Bendarjevskiy, 2011). Highlighting the historical context helps to show the context surrounding the immigrants who arrived to Hungary.

Since the outbreak of war in Ukraine, we do not yet have any data on attitudes towards Russian speakers living in Hungary. Due to the similarity between the Ukrainian and Russian languages, outsiders may easily confuse the two. Besides, the use of the Russian language was natural among Ukrainians prior to the war. In this situation, we are witnessing significant changes and the development of conflicts that will certainly need to be taken into account in the future when investigating the background to the acculturation of Russian speakers.

### Acculturation Processes and Adaptation

The acculturation process comprises the psychological and sociocultural changes that take place in an individual as a result of encountering different cultures (Berry, 2003; Berry & Sam, 2016). Individuals do not acculturate in the same way or in one specific way, and the variations that emerge are known as acculturation strategies (Berry, 1980). These strategies can be described in terms of attitudes towards the individual’s own ethnocultural group and the host society. At individual level, they are made up of two (typically interrelated) components: the individual’s attitudes (i.e., individual preferences regarding acculturation) and their actual behavior as reflected in the intercultural space (Berry, 2005). Based on these components, four possible strategies can be distinguished, based on how the individual cultivates their original ethnocultural traditions, values, and customs in addition to the traditions, values, and customs experienced and learned in the new country. These strategies are (a) integration; (b) separation; (c) assimilation; and (d) marginalization. Research has shown that integration is the most common acculturation strategy and has the most positive outcome in terms of adaptation (Nguyen & Benet-Martínez, 2013). Assimilation and separation strategies also have a good adaptation outcome, as they are coupled with well-being and positive psychological parameters at a practical level (Berry, 2005). The acculturation process plays an important role in mental health, and the integration strategy seems to have the most favorable outcome here as well (Nakash et al., 2012). Among adolescent Russian immigrants living in Israel, integration efforts have been associated with positive psychological and sociocultural outcomes (Eshel & Rosenthal-Sokolov, 2000). At the same time, it is important to mention research and literature in which the relationship between acculturation strategies and adaptation is not considered to be as strong as was previously assumed. A meta-analysis based on longitudinal studies by Bierwiaczonek and Kunst (2021) pointed to a weak correlation between acculturation and adjustment. The positive effect of integration, which was previously believed to be the best strategy from the point of view of psychological parameters, was not corroborated by the statistical background (Bierwiaczonek & Kunst, 2021). For a more in-depth understanding, the use of qualitative research is recommended. Since culture, identity, and human experience are extremely complex, quantitative methods do not allow for a comprehensive understanding of processes related to adaptation, acculturation, and mental health (Wang & Castro, 2016).

#### Acculturation Stress

The acculturation process involves efforts to learn a new lifestyle. These efforts affect the individual’s existing lifestyle and identity and are thus associated with stress (Rudmin, 2003). Furthermore, according to the theory of acculturation strategies, stress arises at the individual level when the individual attempts to diminish the perceived differences between their culture of origin and the dominant culture. Individuals may experience many psychosocial stressors during the acculturation process, such as learning a new language, leaving friends and family members, forming new relationships, and encountering or fearing discrimination. A longitudinal study examining the relationship between acculturation stress and mental health among international students found that the dimension and degree of acculturation stress was a strong predictor of poor mental health and lower psychological quality of life (Taušová et al., 2019). In their examination of the relationship between acculturation strategies and mental health, Berry and Kim (1988) found that integration was accompanied by the lowest level of acculturation stress, followed by the assimilation strategy, while separation and marginalization resulted in the poorest indicators in terms of stress, which also has a negative impact on mental health (Berry & Kim, 1988). Coping strategies are important in terms of dealing with acculturation stress.

### Nonsuicidal Self-injury

Nonsuicidal self-injury (NSSI) can be defined as an episode in which an individual harms themselves without suicidal intent (Nock et al., 2006; Skegg, 2005; Madge et al., 2008; Favazza, 2011). The Diagnostic and Statistical Manual of Mental Disorders, 5 th Edition (DSM-5) lists NSSI as a separate syndrome in the “deserves further study” section (American Psychiatric Association, 2013). Nonsuicidal self-injury occurs in both clinical and non-clinical groups. While the prevalence in the non-clinical population is between 15 and 46% (Lloyd-Richardson et al., 2007), in the clinical population the prevalence is much higher, reaching between 40 and 80% (Jacobson et al., 2008). The overlap between suicidality and NSSI in clinical groups is 70% (Nock et al., 2006) and 50% in the non-clinical population (Muehlenkamp et al., 2012). From an epidemiological point of view, NSSI typically appears in adolescence (Nock & Prinstein, 2004). Research into the causes of NSSI has drawn attention to the fact that the background of NSSI is intrapsychic, and that interpersonal tensions and the social context can play a prominent role (Andover et al., 2012; Favazza, 2011; Klonsky, 2007). Furthermore, NSSI can be interpreted as an attempt at emotion regulation (Victor et al., 2012).

Regarding the relationship between life events and NSSI, recent studies have highlighted the significance of stressful life events, especially stress resulting from interpersonal conflicts (Horváth et al., 2020). Studies conducted in Germany indicated a higher risk of NSSI among adolescent immigrants compared to non-immigrant youth (Plener et al., 2015). A large-scale German study involving 10,000 young people found that adolescents with an immigration background were at greater risk of suicide and self-harming behavior (Donath et al., 2019). In their summary, the researchers emphasized the likelihood of specific reasons behind NSSI and suicidal behavior among immigrants that merit comprehensive investigation using a quantitative method supplemented by qualitative research. This is extremely important from the point of view of designing sufficiently sensitive prevention programs (Donath et al., 2019).

## Objective

In the present study, we focused on adolescents who had experienced NSSI and/or other mental health difficulties, and we investigated their experiences in relation to immigration by means of the following two questions:

1. How adolescents describe their own immigration experience?
2. What stressful experiences or coping strategies emerge during the immigration experience?

## Methods

### Ethical Aspects

Before implementing the study, we obtained the approval of the ethics committee of the Medical Research Council (registration number:). Participants were first given written and oral information about the study and had an opportunity to ask questions. Parents/guardians and young people over the age of 14 then gave their active, written consent to participate in the study. There was no financial incentive for participation.

The questions were asked by trained and experienced professionals, who, if necessary, interrupted or ended the interview and provided professional assistance to the participant. If an emergency situation arose during the interview (e.g., suicidal thoughts or plans), we spoke to the respective participant and their parents and offered immediate professional help. Based on the subjective impression of the interviewer, in the case of the interviews that were selected for analysis the participants talked about their immigration experiences in a state of high emotional arousal. Thus, all of them were offered a break as well as psychological help. One of the participants did start to cry, making a brief interruption to the interview necessary to allow them to regain their composure. Despite this, the participants willingly responded to the questions and reported on their experiences. After the interview, two participants inquired about psychological counseling and received help.

The audio material of the interviews was transcribed verbatim and then deleted. The interviews and test materials were uploaded to a file-protection cloud called Tresorit and connected to a laptop used for work. The password-protected laptop is not accessed by anyone other than the researchers.

### Procedure

To answer our research questions, we conducted interviews with Russian-speaking adolescent immigrants living in Hungary. At our request, the teachers at a bilingual Russian–Hungarian high school in Budapest informed Russian-speaking students with an immigrant background and their parents about participation in the research. After the parents and adolescents had signed the informed consent forms, the interviews were organized at the school. The interviews were conducted on two occasions to avoid placing an excessive strain on the participants. On the first occasion, demographic data were collected, and a semi-structured interview was recorded, while the second occasion took the form of a structured child psychiatric diagnostic interview and a questionnaire measuring NSSI. The interviews took place between November 2018 and March 2019, before the outbreak of the Russian—Ukrainian war. The data collection ended at the outbreak of the COVID pandemic.

### Sample

A total of 14 structured interviews were conducted. The interviews were conducted in a high school and not in a clinical context. Mental health measures indicated that five out of 14 participants showed mental vulnerability. Our study is based on the analysis of the interviews with these five participants.

We decided to focus on the experience of young immigrants currently experiencing a more difficult situation in terms of mental health. We chose to highlight adolescents with mental health difficulties because 1. Mentally vulnerable adolescents can face unique challenges and struggles that differ from those of their peers. Understanding their experiences allows to provide targeted support and interventions. 2. Shedding light on the experiences of mentally vulnerable adolescents helps raise awareness about mental health issues among this specific group. It fosters a more supportive and understanding environment. 3. Understanding the experiences of mentally vulnerable adolescents can help improve their overall quality of life and set them on a path to better long-term outcomes.

The average age of the selected interviewees was 16.2 years (maximum 18 years, minimum 15 years). In terms of gender distribution, there was one boy and four girls.

The names of all the participants have been changed. All the participants lived in a family with both parents and none of them reported the occurrence of any mental disorders in the family. In terms of socioeconomic status, all the selected participants identified their families as having an average financial situation. All participants live in the capital of Hungary—Budapest.

More detailed data on the participants'immigration and mental health can be found in Table 1.

**Table 1:** Participant data: basic data, self-harm questionnaire data, MINI Kid structured interview data. The table contains the data of the participants and the data of the questionnaires (self-harm questionnaire, MINI Kid structured interview). You can read the pseudonym, the age of the adolescent, the age at the time of immigration, the number of occurrences of self-harm and the age of occurrence, the reason/purpose of the self-harm and the type of mental disorder indicated by the MINI Kid structured interview

Name	Age	Age at time of immigration	Appearance of self-injury (DSHI)	Stated reasons for self-injury (DSHI)	Mental health difficulty according to MINI Kid
1. Rita	18	11	2 times between the ages of 13 and 15	Improvement of emotional state	Panic, agoraphobia
2. Boris	15	11	3 times between the ages of 12 and 13	Improvement of emotional state	Suicidal thoughts, depression, alcohol consumption
3. Olga	16	11	Several times between the ages of 12 and 13	Conflict or bad emotional state before self-injury, reduction of tension	Suicidal thoughts
4. Mila	17	15	Once at the age of 15	Anger. She had thought about harming herself several times without actually doing it	Depression, panic
5. Kamilla	15	13	Once, at the age of 14	Improvement of emotional state	Depression

### Measurements

#### Demographic Questionnaire

A demographic questionnaire was compiled specifically for the study, focusing on the following data: participant’s gender, age, parents’ education, economic activity, family composition, siblings, family history of psychiatric disorders, birth information, participant’s psychological/psychiatric treatment, chronic disorders, schooling, and school results. The data sheet was filled out by the participant’s parents/guardians.

#### The Semi-structured Interview

The five selected interviews were all conducted in Russian. The interviews lasted for a minimum of 24:16 min and a maximum of 34:30 min.

The interview questions focused on the participant’s personal experiences, including a general self-presentation, immigration history, and their immersion in their own experience. The interview also included open questions about their identity, as well as questions about the acculturation process. The adaptation process was assessed by asking questions such as “How do you feel?” and “How are you doing?” To investigate perceived similarities and differences and the emotional aspects of identity, we formulated the following questions: “How similar or different do you think you are to an average Hungarian youngster?” and “How similar or different do you think you are to an average teenager from your place of origin?” The same questions were also asked about their family.

The semi-structured interview questions were based on the literature on acculturation and adaptation (Berry, 1997; Berry et al., 1987; Birman, 2006; Phinney, 1990; Schwartz, et al., 2006). We deliberately did not ask about mental health, as we wanted to focus as much as possible on the experience of immigration. We did not ask about their experiences of mental health or how these related to their immigration experiences, as we wanted to avoid interpreting and influencing the adolescents.

#### The Mini International Neuropsychiatric Interview for Children and Adolescents

The Mini International Neuropsychiatric Interview for Children and Adolescents (MINI Kid) is a structured psychiatric diagnostic questionnaire for children aged between 6 and 18 that provides an opportunity to establish a diagnosis according to the DSM-5 (Balázs et al., 2004) and assess subthreshold forms of disorders (Balázs et al., 2004; Sheehan et al., 2010). The Hungarian version of the MINI Kid was adapted by Balázs and her colleagues (Balázs et al., 2004). For the purposes of the present research, we translated the questionnaire into Russian with the permission and cooperation of the original author, David Sheehan (Roszik-Volovik et al., 2020). The participants were able to choose whether the interview was conducted in Hungarian or Russian. The average recording time for the questionnaire was 25 min. In the case of subjects below the age of 13, the interview took place in the presence of a parent, and the parent was able to intervene in the interview at any time, according to the MINI Kid protocol. Children over the age of 13 attended the interview alone. The questionnaire was administered by a trained researcher.

#### Self-injury Questionnaire

The Deliberate Self-Harm Inventory (DSHI) is a 17-item self-report questionnaire that assesses whether an individual has engaged in direct self-injuring behavior, self-injury of the body surface, self-cutting, self-burning, self-biting, or skin damage inflicted by other methods. The DSHI assesses the frequency, severity, and duration of self-injury. The questionnaire uses a branching technique: If the answer to the question about self-harm is “no,” then there are no further questions. However, if at least one of the answers is “yes,” then further questions are asked to ascertain the age at which the self-injury occurred, and the frequency, duration, and severity of self-injury (Gratz, 2001).

### Data Analysis

The interviews were recorded then transcribed verbatim. One author analyzed all five interviews. The analysis was carried out using the interpretative phenomenological analysis (IPA) method described by Smith and his colleagues (Smith et al., 2009). The left-hand margin of the interview transcript contained the emerging topics, while the right-hand margin contained the related exploratory comments. The method makes it possible to explore the participants’ inner experiences and their related meanings based on their narratives.

As a final step, one of the authors carried out an independent check on the IPA analysis. Deviations and comments were discussed together, and corrections were made accordingly.

By using IPA as a research method, we can try to go beyond the statistics and surface-level data, gaining a deeper understanding of the immigrant experience and its implications for mental health.

This content analysis method provides an opportunity to explore the unique and subjective experiences of immigrants that can impact their mental health. It uncovers the various factors that impact their mental well-being, including acculturation, social support, fears, and cultural identity. We find that this method can explore the impact of migration, resettlement, language barriers, and acculturation stress on mental well-being. Delving into participants'narratives, IPA aims to identify stressors and coping mechanisms used by immigrants that may have an impact on mental health. IPA empowers participants to share their stories, amplifying their experiences and needs.

## Results

**Fig. 1:**
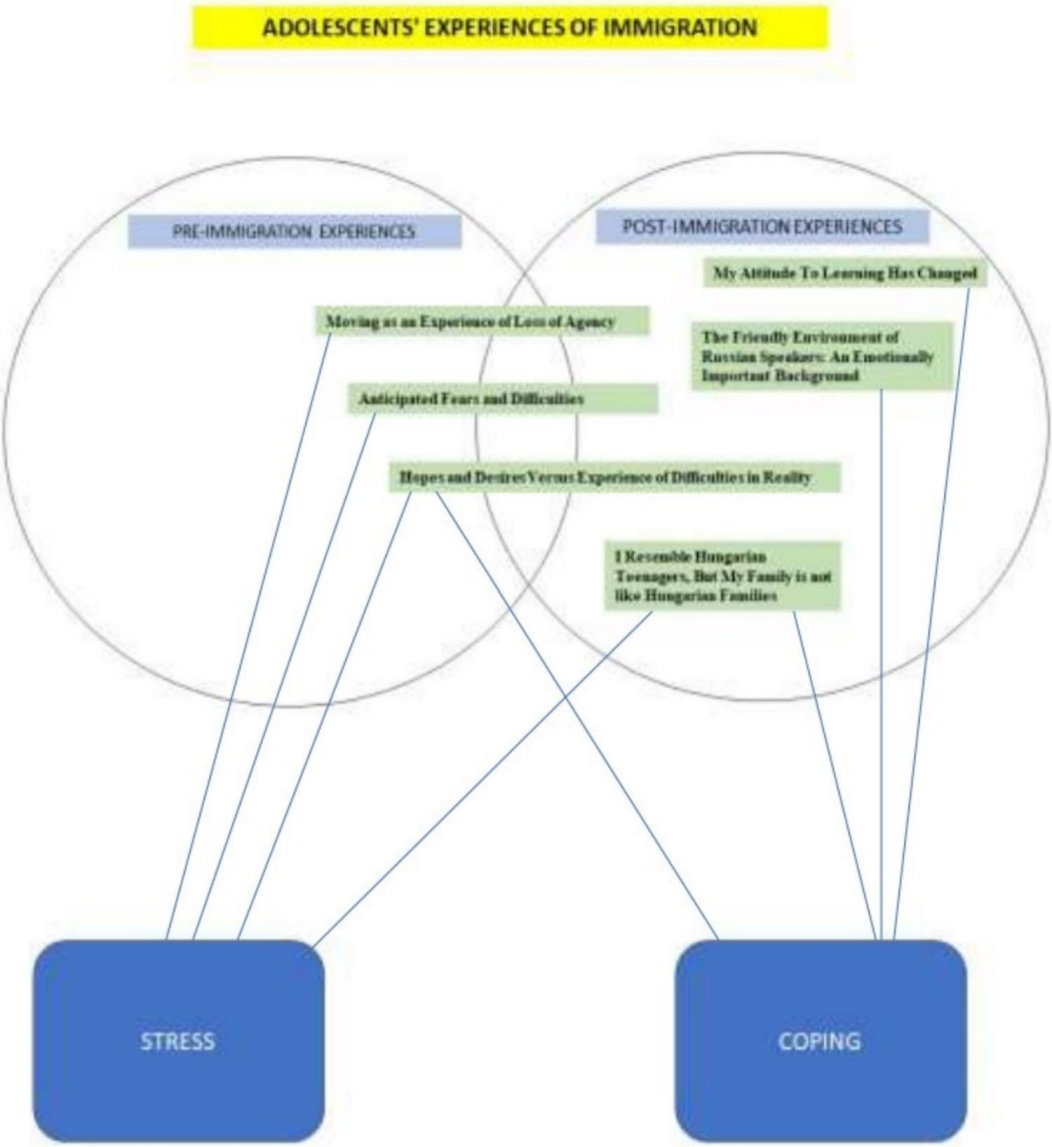
Adolescents' experiences of immigration. Display of the six themes over time (from pre-immigration to post-immigration). It can also be seen whether the given themes is related to the source of stress or an attempt to cope

The presented themes highlight the stressors faced by immigrant adolescents and their ways of coping. The figure illustrates how immigration can take its toll on adolescents'mental functioning, potentially placing a psychological strain on mental health and increasing mental health vulnerability. The figure shows that the process starts in the pre-immigration period and then persists over a long period of time during the process of integration and acculturation. This temporal circumstance further emphasizes the strain on mental health. Although not the primary focus of our study, it is conceivable that a causal stress-coping effect mechanism may exist Fig. 1.

In two subjects, both stressors and coping appear at the same time. Thus, in the case of Hopes and Desires Versus Experience of Reality, the hope for a better life serves as a coping mechanism, but it is eventually replaced by the harsh reality, resulting in a stressful experience. Another theme, We Are Similar on a Personal Level, But Different on a Family Level, reveals that in terms of self-perception and social comparison, seeing themselves as similar to Hungarian peers gives immigrant adolescents a reassuring and comforting feeling, but they encounter stress due to perceiving difference at the family level. The last theme explores the possibility of connecting with Russian speakers as a coping mechanism, while also highlighting the challenge of connecting with Hungarian speakers due to language barriers, which can be a potential source of stress.

Detailed description of the themes:

### Moving as an Experience of Loss of Agency

The decision to move and the choice of destination country were made by the parents. The adolescents were not involved in these decisions and had little or no knowledge about their future country. Loss of control appeared at both the cognitive and emotional level. At the same time, there was evidence of feelings of helplessness. The exclusion of an individual’s will can be regarded as a potentially stress-inducing situation.

The following quotation is taken from a 15-year-old boy’s recollection of his feelings on finding out that he was going to move to Hungary. His experience reflects his lack of knowledge and control in a situation in which the question of his own will did not even arise:

*My parents said that the situation in Russia is not very stable, for example in terms of education (…). They said we are going to Europe, to Hungary, and I asked what is Hungary? I had no special role in this situation, they said I would go, and that was it.* (Boris, 15 years old)

A 16-year-old girl also pointed out that it was her parents’ decision to move. After moving, she had to stay alone without her family for months.

*We had been planning to move for a very long time. In other words, my parents planned it (...) they left me here alone while they went back to get the stuff. A good friend of ours looked after me. After about six months, my parents came back.*

(Olga, 16 years old)

### Hopes and Desires Versus Experience of Difficulties in Reality

The interviews revealed how the adolescents began to develop positive ideas about moving. Compared to what they had imagined, however, their encounter with reality resulted in disappointment. The adolescents found themselves isolated and lonely, in a life situation without friendly relationships. The time spent studying and their inability to speak the language were additional obstacles, besides the absence of real relationships.

While 16-year-old Olga had initially been enthusiastic and hopeful for positive changes, it turned out that she did not in fact have time to develop relationships. Her lack of free time was genuine, as she had to study intensively so as to be able to keep up with her Hungarian contemporaries.

*In the beginning, there was joy in me that it was good, something new in life. How cool, we're going to Hungary. Well, we've arrived. In the beginning, I studied a lot and didn't have time to socialize.* (Olga, 16 years old)

Hopes for a positive, better life were present at the cognitive level, although the real difficulties could not be anticipated. The following quotation is taken from 18-year-old Rita’s recollection and interpretation of her early days in Hungary:

*I knew it would be better for me here, but it was very difficult at the beginning. (...) I had no friends, and I had a partner before moving. To this day, I don't get along with my classmates, and of course, the language is also difficult.* (...)

*With the move, I had to face unexpected situations that caused a strong feeling of uncertainty and caused long-term stress. They were looking for a school for me for a long time. You'd think that's what teenagers need, another summer break. But I lived under stress of what if they wouldn't admit me anywhere.* (Rita, 18 years old)

In reality, following immigration the adolescents soon experienced loneliness and isolation. They were faced with the fact of being left without friendships, either due to their initial school adaptation or their living circumstances:

*When we first arrived here, I was forced to go to primary school. I didn't finish 8 th grade, I went to language lessons, I didn't have any friends.* (Kamilla, 15 years old)

The following quotation is taken from Boris’s recollections of the initial period, when he experienced loneliness and isolation and became conscious of his marginality:

*We moved to a big house on the outskirts of town at the beginning. This is very far from the center. It took two hours to get to the center by car because of traffic jams. I didn't have friends then or I couldn't meet them because of the long distance. So, I just sat at home and looked out the window or played with the dog.* (Boris, 15 years old)

### Anticipated Fears and Difficulties

The young people experienced anticipatory fears in relation to social situations. They were afraid of not being treated as equals, for example, and of becoming objects of ridicule. Such fears were anxiety states that may have been manifested somatically. Fear affected the interviewees’ self-concept and was associated with feelings of insecurity. Language appeared to play a decisive role in terms of the interviewees making sense of how others saw them and of their position among their peers:

*My biggest fear is that they will treat me like a toy parrot who is learning words. Now they treat me as a human being, and not as someone who came here and doesn't know Hungarian.* (Boris, 15 years old)

Mila reported feeling a sense of shame in social situations due to not knowing the language. However, a friendly approach on the part of her peers made a difference:

*When they correct me, I start to feel ashamed, my face turns red. But if they do it in a friendly and kind way, I always learn from how they correct me.* (Mila, 17 years old)

Kamilla described her initial fears in social situations, which arose due to feeling different from others because of her background as an immigrant. However, this feeling had changed over time:

*At first, I felt that I was not like the others, and I had the feeling that they looked at me strangely, but now that I am talking to them, I don't even notice that I am different.* (Kamilla, 15 years old)

Boris was able to maintain a comfortable sense of balance by separating his social spaces, which prevented him from being overwhelmed by his perceived loss of control. His efforts indicate the difficulties involved in maintaining a sense of social competence, which is important among young people:

*For me, friends, family, and private life are separate spaces, I would never mix them up. The only event where they would all meet would be my funeral. I love everyone very much, but it's better for me if everyone is separate.* (Boris, 15 years old)

Starting to communicate is a difficult and stressful event in itself:

*…for me, starting to communicate in a new situation is extremely difficult.* (Rita, 18 years old)

#### Resemble Hungarian Teenagers, But My Family is not like Hungarian Families

A shared experience among the young people was their realization that, besides their immigration-related difficulties, they faced general difficulties similar to those experienced by Hungarian youngsters. They were able to connect with young Hungarians through these experiences. On the other hand, their family experience differed from that of the average Hungarian family, and such differences were not eliminated by the move to Hungary.

The youngsters’ experiences demonstrated that all Russian-speaking and Hungarian young people face similar difficulties, with minor differences. Once they were living in a similar situation to their Hungarian peers, for example, they struggled in a similar way with their studies or experienced similar difficulties in relationships. However, from a family perspective, the young people reported experiencing differences arising from immigration and cultural differences as a family.

Boris reported not feeling any different from his Hungarian-speaking adolescent contemporaries, although he had experienced changes at the family level, in which the new environment had become important:

*I wouldn't say I'm particularly different. Maybe it's just the mentality that Hungary is Europe, but teenagers these days are very similar to Russians. Let's say, for example, in Russia it's not appropriate to blow your nose in class or on the street, here they think it's better to blow than to sniff. But there are nihilists here and there, and I am one of them. That's why I wouldn't say that the difference between us is big (...) It's not the family that differs, but the environment. We have more opportunities here.* (Boris, 15 years old)

Rita essentially experienced similarities with her peers. In her case, a feeling of difference arose due to her family’s specific situation, which was accompanied by an interpretation of the resulting conflict:

*I'm no different from Hungarians (…) My family is different from average Hungarian families, but probably from all families, because the father doesn't work here and because of this we have quite a lot of arguments in the family.* (Rita, 18 years old)

In Kamilla’s experience, at a personal level she was no different from her Hungarian-speaking peers, although at a family level, the situation was different:

*I am no different from the Hungarians. There is no cultural difference either (...). My family is different because our customs are different. We are Pravoslavs.*^2^ (Kamilla, 15 years old)

For 16-year-old Olga, the experience of difference was related to differences in shared childhood experiences, which can be important in social situations. In addition, the experience of family-level difference also emerged:

*In Russia, children are brought up differently. There they sing those songs in the kindergarten, here in the kindergarten they sing other songs. And there are very famous old cartoons, I know them very well. There are cartoons here too, only they are in Hungarian, they are Hungarian cartoons. Our childhood experiences are different. Children are raised differently in Russia. (…) My mother is Russian, in the family we sometimes speak Russian and sometimes Hungarian. (*Olga, 16 years old)

### My Attitude To Learning Has Changed

In all cases, immigration had been undertaken in the hope of a better life. The parents’ considerations involved better prospects and job opportunities, which the adolescents integrated into their own narrative. This perspective prompted a change in the adolescents approach to learning. Their sense of responsibility was strengthened and their thinking matured. The future became something that the adolescents could have control over, which was associated with self-confidence and growth potential. They discovered that they could assert themselves with the help of learning.

Boris described how experiencing opportunities strengthened his desire to learn:

*When you go to another country, you see better opportunities, even I felt like doing it. I understand that I will try hard towards the end of school and later on as well. There (in Russia), good knowledge is destroyed by the environment. In other words, the environment has a very strong influence, so people don't try hard enough.* (Boris, 15 years old)

Rita also discovered the value of learning. Immigration had confronted her with her own knowledge, which changed her attitude to learning:

*I became more independent in Hungary. This is where I realized I had to do everything myself. I became more responsible. I feel good at school now. There (before moving), my parents had expectations of what kind of grades I should have. Not here anymore. I realized that I had no knowledge, that my previous strategy was wrong, that I was paying attention to marks and not to knowledge.* (Rita, 18 years old)

Olga realized that she could achieve her goals through language learning:

*When we arrived here, I realized that ok, I have to learn the language if I want to live here.* (Olga, 16 years old)

Mila also described the experience of being given a chance:

*I have to study here because I was given such a chance to be abroad and to make it better for me in the future.* (Mila, 17 years old)

### The Friendly Environment of Russian Speakers: An Emotionally Important Background

The young people had experienced the fact that Russian-speaking friends provided emotionally important social experiences and compensated for the sense of absence. This situation was also related to language difficulties.

Boris described how much he missed his Russian-speaking friends:

*I miss my Russian friends and see them on Fridays.* (Boris, 15 years old)

Due to the language barrier, social communication was easier with Russian speakers, and also associated with a kind of emotional background. The young people expressed a wish to be able to communicate with Hungarian speakers in the same way, which was not yet happening:

*I notice that it is easier for me to communicate with Russian speakers. This makes me a little sad. Sometimes I feel some kind of language barrier. I feel that I communicate with Hungarians in a different way. I would like to somehow overcome this barrier.* (Rita, 18 years old)

*I don't speak Hungarian very well, so I mostly have Russian-speaking friends.* (Kamilla, 15 years old)

The youngsters described times when it was easier to join already established social situations in a Russian-speaking environment:

*I am no different from them (Hungarians), I have met people who accept me. But there were already established cliques in my class, and I teamed up with the Russian speakers.* (Mila, 17 years old)

## Discussion

To the best of our knowledge, the present study is the first to examine the immigration-related experiences of Russian-speaking adolescents who had experienced mental health difficulties. The IPA analysis revealed six main themes that highlighted potentially stressful experiences and ways of coping. The qualitative research method gives us the opportunity to listen and better understand young people's experiences of immigration. This allows us to understand their difficulties in a more nuanced way.

If we extract the first theme the"Moving as an Experience of Loss of Agency"We can see that the first period after immigration, when the adolescents faced real difficulties, was a sensitive period in terms of mental health since our interviewees reported a deterioration in quality of life during this time. They experienced the breakup of existing relationships and found themselves in a lonely, isolated situation. According to anthropologist van Gennep’s 1960 book *The Rites of Passage*, during major changes in life a person undergoes three experiences: leaving the old; being on the way; and arriving. The adolescents in the present study described abandonment and being on the road, although arrival was still missing. The experience of being abandoned is also accompanied by “uprooting” stress, which arises from the experience of rootlessness (Szabó et al., 2016).

In the initial period, the formation of hopes can also be interpreted as an attempt to cope with stress and the feeling of being out of control which has appeared in the following theme -"Hopes and Desires Versus Experience of Difficulties in Reality". This theme"Anticipated Fears and Difficulties"shows fears are related to feelings of shame in social situations. In several studies, shame has been shown to play a role in mental disorders, while chronic shame can be associated with self-harm and suicidal thoughts (Averill et al., 2002; Brown et al., 2009, Sheehy et al., 2019). A sense of shame can also be a hindering factor when it comes to initiating social interactions, and a great deal of effort is needed to overcome it. The young people in the study experienced immigration and their own cultural differences as negative and as indicative of deficiency. This may be related to the fact that they came from an environment in which there is no tradition of valuing diversity.

Belonging is a basic need, and lack of belonging can have a negative impact on mental health and quality of life (Baumeister & Leary, 1995). Part of the normative crisis is for adolescentse to find their place in social life. In general, the adolescents in the present study considered and wished themselves to be similar to their Hungarian contemporaries and did not feel a great cultural distance. Interestingly, the cultural distance was perceived to be greater from a family perspective, indicating the acculturation gap between the individual and the family levels. Family support is very important, and a supportive family background plays a protective role in mental health. This finding thus raises the question of whether it is worth considering the possibility of involving the whole family when planning support and prevention sessions, thus creating more communication interfaces for young people in this area as well.

Considering the following theme"My Attitude To Learning Has Changed"the pursuit of learning can also be interpreted as a psychological struggle that contains positive potential. Due to their lack of knowledge of the Hungarian language, it was easier for the young people in the study to communicate with Russian-speaking peers, which also had positive psychological effects and helped them remain emotionally balanced. Out of the four acculturation strategies as mentioned by Berry and Kim (1988), our interviewees reported on separation accompanied by acculturative stress and mental health vulnerability as a typical acculturation orientation, even though they wanted to connect with their Hungarian peers. Not integration, rather assimilation appeared as the desired state. This may stem from their previously mentioned ambivalence regarding diversity, which they had brought with them from their country of origin. Our study contributes to the literature debate about the connection between acculturation strategies and adaptational outcomes of immigrant adolescents at a specific point. It reveals that if adolescents perceived during the pre-immigration stage that cultural diversity is not valued in their country of origin, they tend to view their own cultural and language differences in the host country negatively, seeing them as indicative of deficiency. Consequently, in their case, assimilation—where they focus on learning the new culture, blending in with the host culture—is the desired acculturation orientation and strategy. This approach is reassuring for them and associated with positive outcomes, rather than integration, which involves maintaining their culture of origin while also learning about and connecting with the new culture. The cultural norms and values from the home country can shape young immigrants'behaviors, attitudes, and expectations in their new environment. The adolescents may encounter challenges in reconciling their cultural values with those of the host society, leading to acculturation stress and identity conflicts (Sirin et al., 2013; Tartakovsky, 2007).

Our findings reveal that adolescents are exposed to many stressful events during the immigration and adaptation process, which can lead to emotional conflicts which starts in the pre-immigration period. In addition to experiencing a loss of control, disappointment, and loneliness, the youngsters in the present study also found it difficult to engage in meaningful social situations. The need to deal with shame and ambivalent feelings emerged in relation to engagement in social situations. When planning culturally sensitive prevention programs, in addition to considering the involvement of the family it may be important to pay attention to the internal, emotional conflicts and projected fears of adolescent in relation to their immigration and adaptation. The study highlights the significance of language in the migration experience. Policies should prioritize language education and support for immigrant adolescents to improve their communication abilities. Language plays a crucial role in shaping various aspects of the immigrant experience and can have a profound impact on mental health in several ways. Immigrants who do not speak the language of their host country fluently may face communication barriers in various aspects of their lives, such as accessing healthcare, education and social services. These barriers can lead to feelings of frustration, isolation, and potentially contribute to mental health issues. While not explicitly emphasized in existing literature, our findings reveal that for adolescents who strongly desire to belong, having limited fluency in the host language can directly lead to feelings of shame, thereby amplifying their vulnerability to mental health issues.

Language is an important aspect of cultural identity (Ferdman & Horenczyk, 2000; Phinney, et al., 2001; Portes & Schauffler, 1994). Immigrants may experience acculturation stress when trying to adapt to a new language and cultural norms, this fear appeared during the study. Struggling to communicate effectively or feeling a loss of language fluency from their home country can create stress and have an impact on the mental health.

Mental health services should be culturally sensitive, recognizing the unique stressors and coping mechanisms related to their cultural background. Understanding that the process of mental strain starts in the pre-immigration period emphasizes the importance of early intervention and prevention strategies. Providing support and resources for adolescents and their families before and during migration can help mitigate potential mental health challenges. In this way, for example, it is possible to create informative materials that can be made available on the Internet. It is important that they can be made available in several languages, taking into account the language of the target audience. Further research may be aimed at a better understanding of the mechanism of the stress-coping effect. Furthermore, to understand the background and significance of the development of the feeling of shame during the immigration experiences of adolescents.

## Brief outlook in connection with other research

Similar difficulties can be observed when comparing the results of qualitative and quantitative research in other countries and with immigrant groups. For example, the role of the family is very important for the well-being of immigrant youth (Conway et al., 2020, Suárez-Orozco et al., 2011). Based on the literature, as discussed above, we know that family gaps in acculturation are developing, which are stressful for young people (Telzer, 2010; Lim, et al. 2009). Our study also shows that perceptions of one's own family also differ when young people compare them to host country families, as is also evident in Birman and Poff (2011) study of other immigrant groups. Language barriers are also a prominent difficulty for immigrants elsewhere in their integration process and have an impact on their mental health (Potochnick & Perreira, 2010). The experience of losing control that comes with immigration is also a stressful experience for young people, as research in other countries has shown (Kovacev & Shute, 2004). Overall, our findings are consistent with other research, but provide a more nuanced and deeper understanding of the difficulties young people experience, which is particularly acute for young people with mental health problems.

## Limitations

The present study has limitations typical of studies employing the IPA methodology. The sample is small and homogeneous, and the results cannot be used to infer causal relationships and effects. Since several languages were used during the investigation and preparation of the study (Russian, Hungarian, and English), the inevitable language differences can also be regarded as a limitation. We attempted to mitigate these differences during the investigation and preparation of the study within the framework of joint discussions. During the semi-structured interview, we did not directly ask questions related to mental health, but a separate semi-structured interview could have been created for the selected participants. This would have helped to go into the experiences of immigrant adolescents in more detail and to listen to their perspectives on how, in their opinion, immigration affected their mental health. Furthermore, it could have been asked in more detail whether the self-injurious events were connected to their negative immigration experiences. These limitations should be taken into account when planning new research. A limitation of our study design is that our study did not take place in a clinical environment, but in a high school. From the point of view of planning future research, examination in a clinical setting will presumably be able to provide a more nuanced picture of the relationship between the immigration experience and mental health. Furthermore, it is important to take into account the limitation that the terms integration, migrant and immigrant are often defined broadly or with different definitions in the literature, which has an impact on the discussion of complex issues related to mental health.

In summary, we would like to highlight that stressors affecting the mental health of young immigrants are embedded in their country of origin and personal and cultural environment. Understanding the circumstances and the context of immigration contributes to the planning of the most effective prevention programs. This complexity is presumably also valid in the case of adolescents from other cultural environments and other immigration backgrounds. In the present study, for example, attitudes in the country of origin towards cultural diversity were found to be important. This may be significant when planning prevention programs aimed at improving the mental health and quality of life of immigrants. By taking context into account, it becomes possible to offer multifaceted emotional support.

In the present study, country of origin was not taken into account when examining Russian-speaking adolescents. Since the outbreak of war in Ukraine, Russian language users may be experiencing a variety of related difficulties. Depending on origin, language can take on a political color. We assume that the projection of prejudices has intensified regardless of which former Soviet Union country the young immigrants came from. We consider the exploration of this issue to be essential for the planning of targeted programs promoting integration.

## Data Availability

Data used in the research such as interviews and the data of the MINI Kid can not be available due to ethical restrictions. Due to ethical supporting the data are protected by confidentiality.

## Footnotes

1 In our study, we primarily use the concept of integration in accordance with Berry's concept. At the same time, we draw attention to the fact that the studies we refer to may use the concept differently, but in any case they refer to a phenomenon where individuals are able to integrate their previous cultural experiences with the experiences of the new environment. We reflect on this phenomenon in the limitations section.

2 The term"Pravoslav"is an alternate spelling of"Pravoslavie", which is the Russian word for"Orthodoxy"or"Orthodox."It refers to the Eastern Orthodox Christian faith, which is one of the major branches of Christianity and has a strong presence in Eastern Europe and parts of the Middle East.
